# Supplementary material for: A functional assay for serum detection of antibodies against SARS‐CoV‐2 nucleoprotein
Source: EMBO J. 2021 Jul 29;40(17):e108588. doi: 10.15252/embj.2021108588 (PMC8408615; doi:10.15252/embj.2021108588)
Supplement: Supplementary file 2 — Appendix [file EMBJ-40-e108588-s006.pdf]

Appendix

A functional assay for SARS-CoV-2 N-antibodies

Anna Albecka, Dean Clift, Marina Vaysburd, Tyler Rhinesmith, Sarah L Caddy, David M Favara, Helen E Baxendale, Leo C James

Table of contents

Appendix Figure S1………………………………………..…………….……….…...1

Appendix Figure S2 ……………………………………..…………….……….…..3

Appendix Figure S3 ……………………………………..…………….……….…..6

Appendix Figure S4 ……………………………………..…………….……….…..7

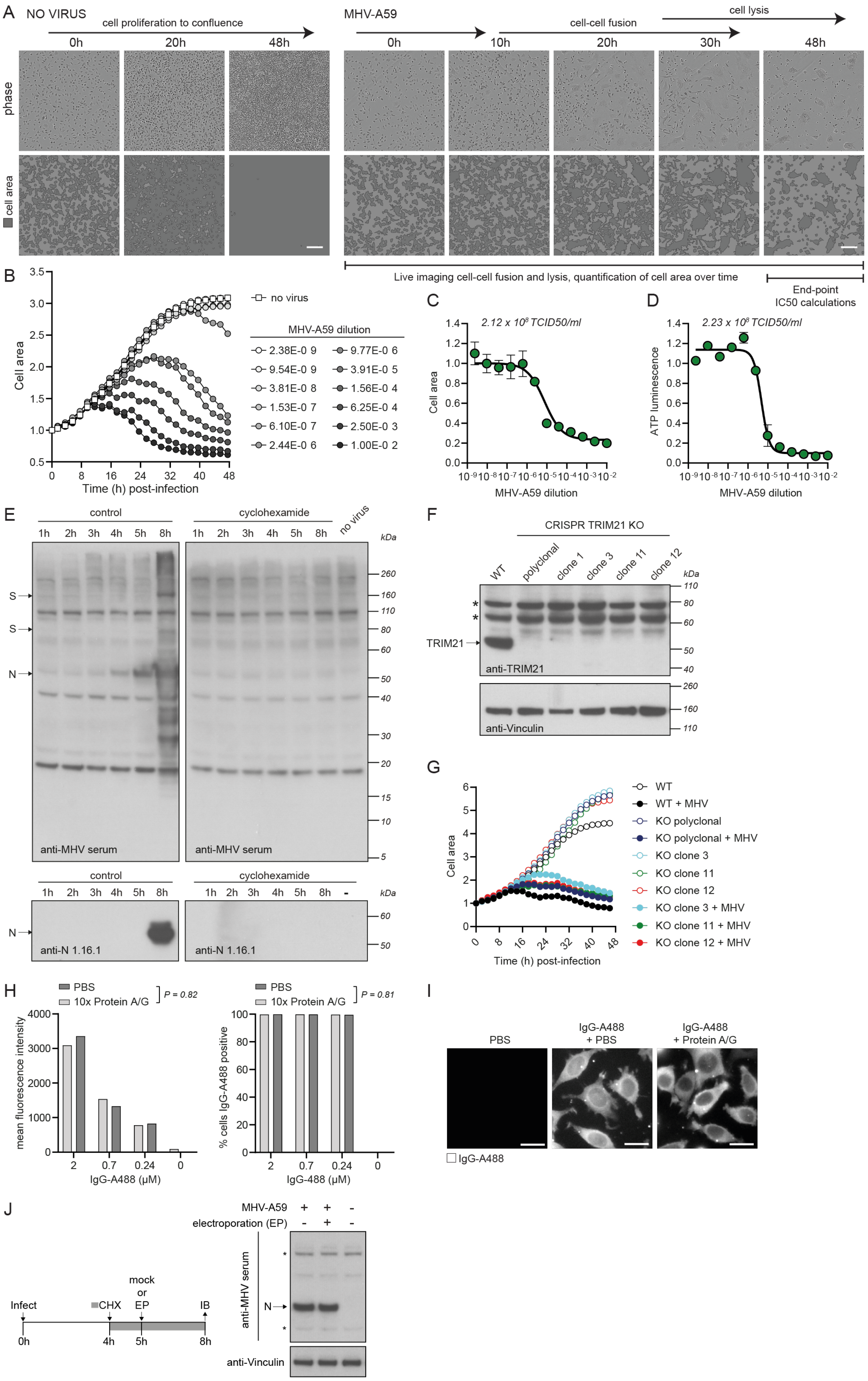
A
NO VIRUS
cell proliferation to confluence
0h
20h
48h
MHV-A59
0h
10h
cell-cell fusion
20h
30h
cell lysis
48h
phase
cell area
Live imaging cell-cell fusion and lysis, quantification of cell area over time
End-point IC50 calculations
B
Cell area
Time (h) post-infection
no virus
MHV-A59 dilution
2.38E-0 9
9.54E-0 9
3.81E-0 8
1.53E-0 7
6.10E-0 7
2.44E-0 6
9.77E-0 6
3.91E-0 5
1.56E-0 4
6.25E-0 4
2.50E-0 3
1.00E-0 2
C
2.12 x 10⁸ TCID50/ml
Cell area
MHV-A59 dilution
D
2.23 x 10⁸ TCID50/ml
ATP luminescence
MHV-A59 dilution
E
control
cyclohexamide
no virus
1h 2h 3h 4h 5h 8h
kDa
S
S
N
anti-MHV serum
anti-N 1.16.1
F
CRISPR TRIM21 KO
WT
polyclonal
clone 1
clone 3
clone 11
clone 12
TRIM21
anti-TRIM21
anti-Vinculin
G
Cell area
Time (h) post-infection
WT
WT + MHV
KO polyclonal
KO polyclonal + MHV
KO clone 3
KO clone 11
KO clone 12
KO clone 3 + MHV
KO clone 11 + MHV
KO clone 12 + MHV
H
PBS
10x Protein A/G
P = 0.82
P = 0.81
mean fluorescence intensity
% cells IgG-A488 positive
IgG-A488 (μM)
I
PBS
IgG-A488 + PBS
IgG-A488 + Protein A/G
IgG-A488
J
Infect
CHX
mock or EP
IB
0h
4h
5h
8h
MHV-A59
electroporation (EP)
anti-MHV serum
N
anti-Vinculin

Appendix Figure S1: Kinetics of MHV-A59 infection can be determined using live cell microscopy. (A) Time-course of L929 cells either mock infected or infected with MHV-A59, showing either phase contrast (upper row) and fitted cell area (lower row, darker grey). Scale bar = 200µm. **(B)** Quantification of cell area at different timepoints post-infection with a titration of viral doses. **(C)** Cell area at 48h post-infection at a range of MHV-A59 doses, fitted with a non-linear regression curve to calculate a TCID50 value. **(D)** Same as (C), except using ATP luminescence instead of cell area. **(E)** Western blot at different timepoints post-infection with MHV-A59 using anti-MHV polyclonal antisera or anti-N monoclonal antibody 1.16.1. Note the increase in viral proteins, particularly S and N, by 8h post-infection. Treatment with cyclohexamide to prevent protein expression demonstrates that this increase is the result of de novo protein synthesis. **(F)** Western-blot of L929 cells that have undergone CRISPR-Cas9 for TRIM21 knockout. Both polyclonal cells and clones show loss of TRIM21 protein. The presence of non-specific bands detected by the anti-TRIM21 antibody is indicated by an asterisk. **(G)** Growth of WT and TRIM21 KO cells in the presence (filled circles) or absence (empty circles) of MHV-A59. **(H)** L929 cells were electroporated with different concentrations of fluorescent antibody (IgG-A488) in the absence (PBS) or presence of 10x antibody concentration of Protein A/G and analysed 1h later by flow cytometry. **(I)** Microscopy images of cells from (H) at 6h post-electroporation. Scale bar = 20µm. Protein A/G does not interfere with cytosolic delivery of antibody. Data was analysed using a variable slope nonlinear fit (C&D) or a Students *t*-test (H). Error bars depict the mean +/- SEM. All data represents at least two independent replicates. **(J)** L929 Cells were infected with MHV-A59 for 4h, then cyclohexamide (CHX) was added to block further viral protein synthesis. After 5h, cells were electroporated with buffer and left for a further 3h before being western blotted for cellular N protein levels (*denotes a non-specific band).

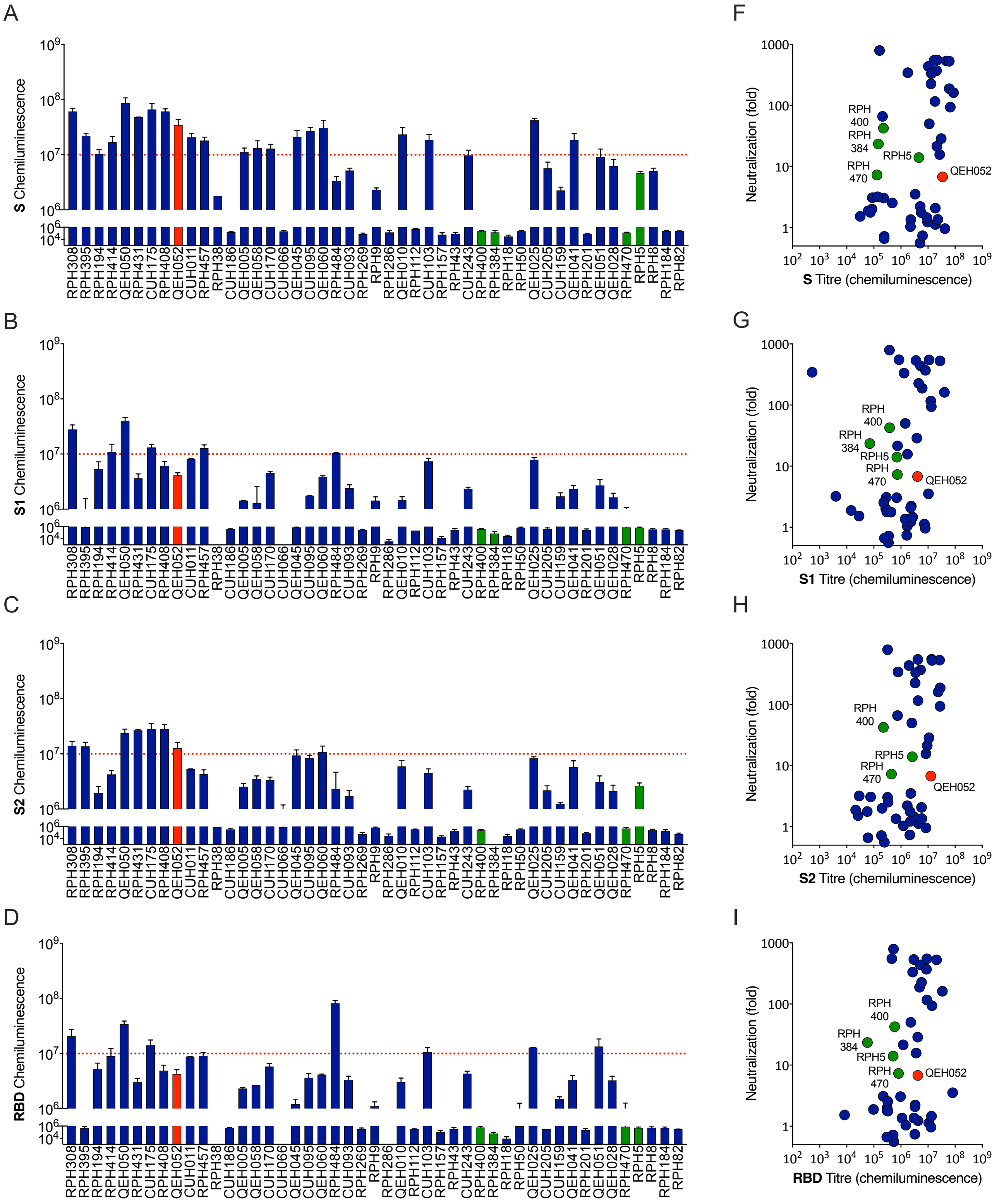

Appendix Figure S2: Specific antibody levels in SARS-CoV-2 convalescent sera and correlation with intracellular neutralization activity. (A-D) Capillary-based protein

detection (Jess) of antibodies against SARS-CoV-2 antigens in convalescent sera, quantified by chemiluminescence. **(F-I)** Correlation between intracellular neutralization (fold decrease in SARS-CoV-2 replication), as quantified by EDNA, and antibody titre, as quantified by chemiluminescence by Jess. Error bars depict the mean +/- SEM. All data represents at least three independent replicates.

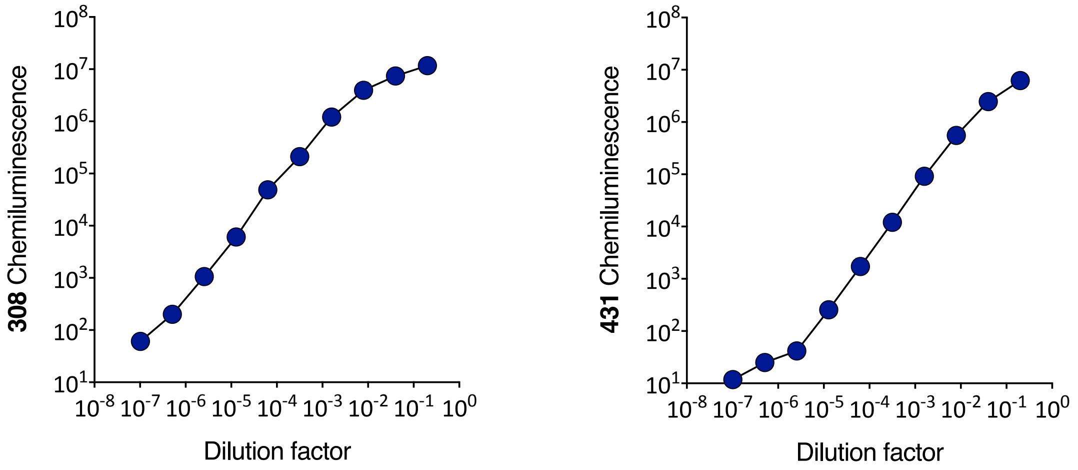

Appendix Figure S3: Quantification of anti-N chemiluminescent signal in a titration of serum from two SARS-CoV-2 convalescents. Jess is able to detect the presence of N-antibodies in serum that is diluted > million-fold.

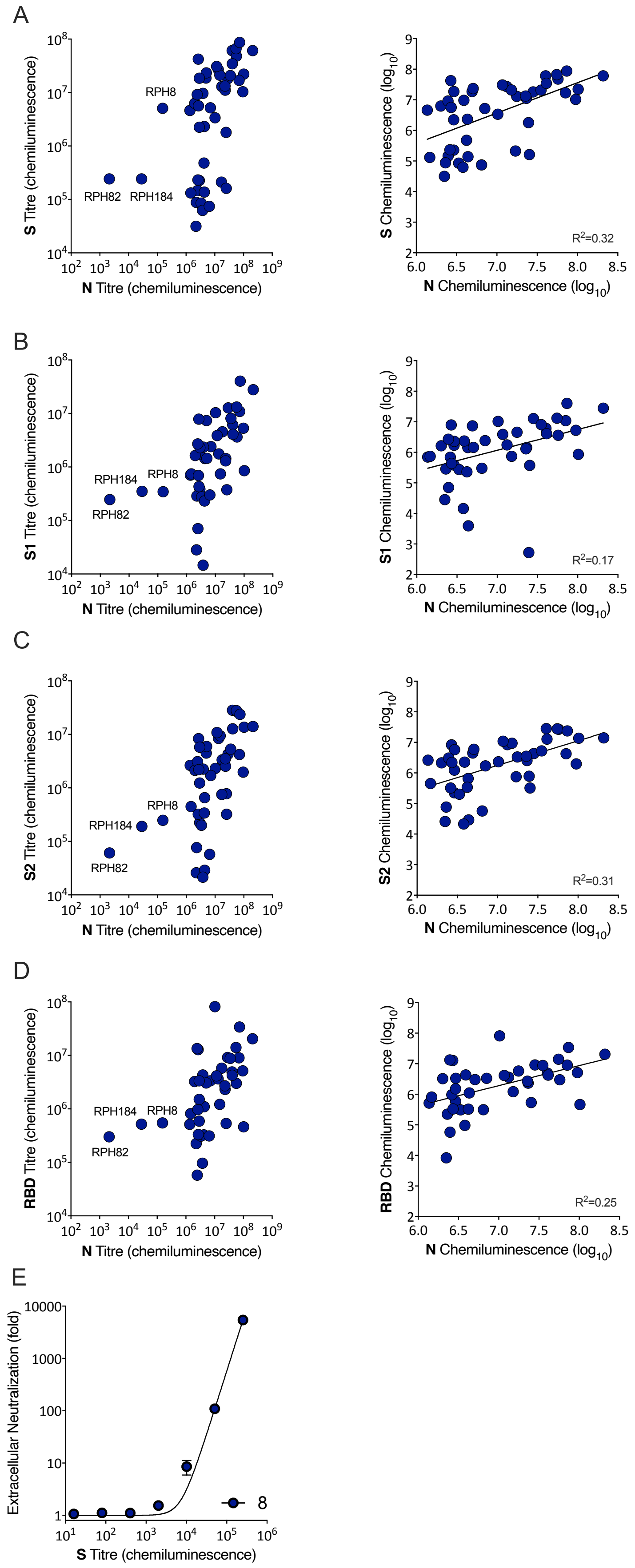

A
S Titre (chemiluminescence)
N Titre (chemiluminescence)
RPH8
RPH82
RPH184
S Chemiluminescence (log10)
N Chemiluminescence (log10)
R2=0.32
B
S1 Titre (chemiluminescence)
N Titre (chemiluminescence)
RPH184
RPH8
RPH82
S1 Chemiluminescence (log10)
N Chemiluminescence (log10)
R2=0.17
C
S2 Titre (chemiluminescence)
N Titre (chemiluminescence)
RPH8
RPH184
RPH82
S2 Chemiluminescence (log10)
N Chemiluminescence (log10)
R2=0.31
D
RBD Titre (chemiluminescence)
N Titre (chemiluminescence)
RPH184
RPH8
RPH82
RBD Chemiluminescence (log10)
N Chemiluminescence (log10)
R2=0.25
E
Extracellular Neutralization (fold)
S Titre (chemiluminescence)
8

Appendix Figure S4: Correlation between N-antibody levels and antibodies against other SARS-CoV-2 antigens in convalescent sera. (A-D) Levels of antibodies against different antigens in individual sera, as quantified by chemiluminescence. In each case, levels are plotted against the N-antibody titre. Both raw and log10-transformed data are shown, with the R^2 correlation coefficient calculated in each case in the absence of outliers RPH8, RPH82 and RPH184. **(E)** Extracellular neutralization (ie no electroporation) of a titration of strongly S positive but weakly N positive serum. Data was analysed using a linear regression.
