# Supplementary material for: A functional assay for serum detection of antibodies against SARS‐CoV‐2 nucleoprotein
Source: EMBO J. 2021 Jul 29;40(17):e108588. doi: 10.15252/embj.2021108588 (PMC8408615; doi:10.15252/embj.2021108588)
Supplement: Supplementary file 3 — Movie EV1 [file EMBJ-40-e108588-s005.zip › MovieEV1/Movie EV1 legend.docx]

**Movie EV1:** Live phase contrast microscopy of L929 cells in the presence or absence of MHV-A59 infection.
