# Supplementary material for: A functional assay for serum detection of antibodies against SARS‐CoV‐2 nucleoprotein
Source: EMBO J. 2021 Jul 29;40(17):e108588. doi: 10.15252/embj.2021108588 (PMC8408615; doi:10.15252/embj.2021108588)

Appendix Figure S1 source data

uncropped blots for panel E

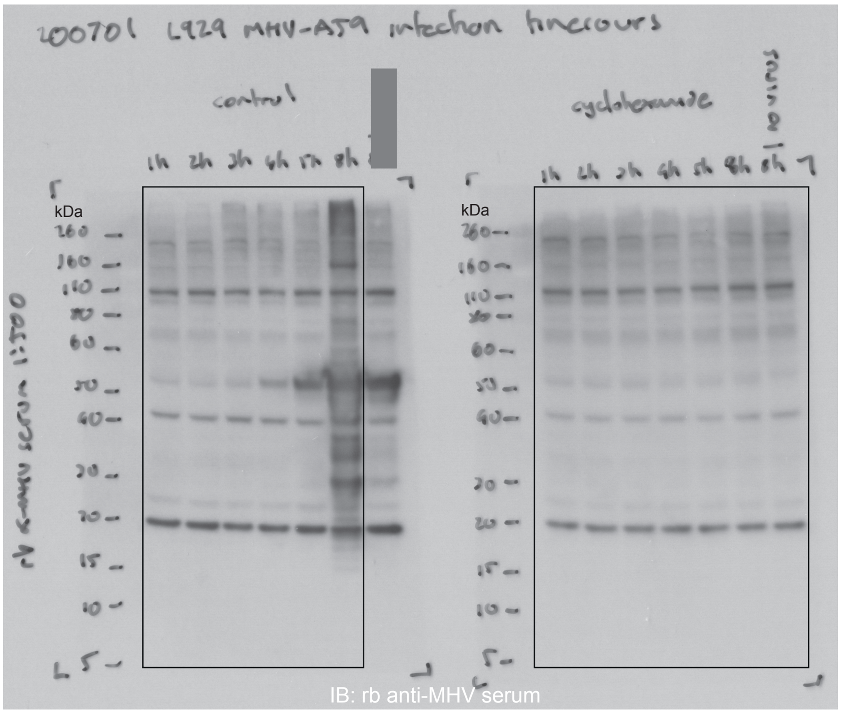

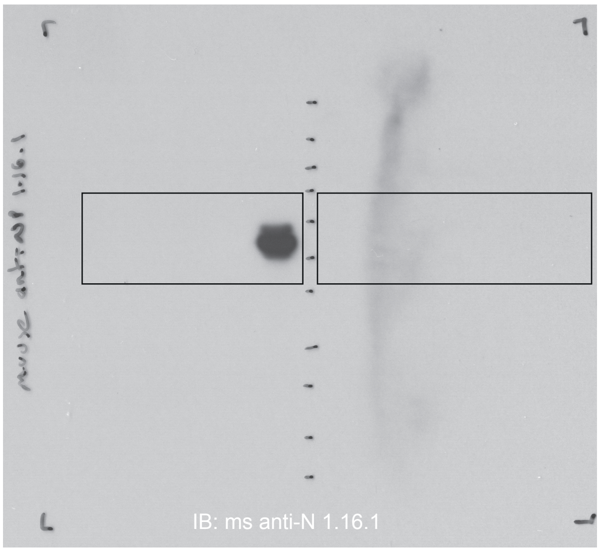

uncropped blots for panel F

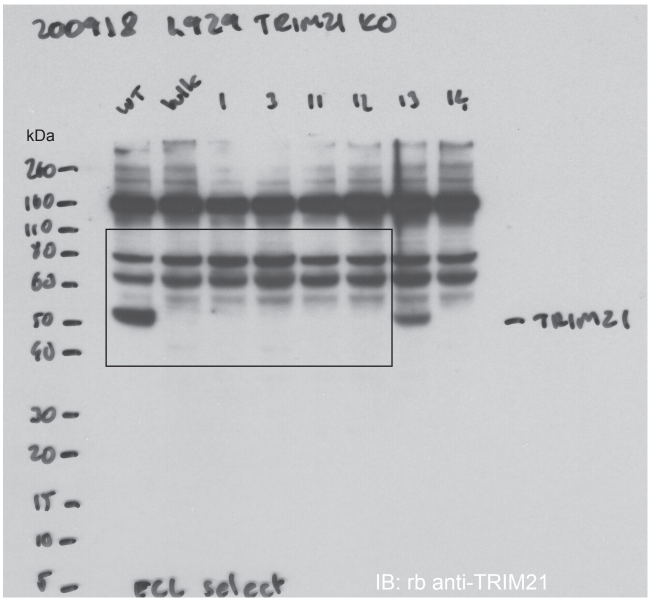

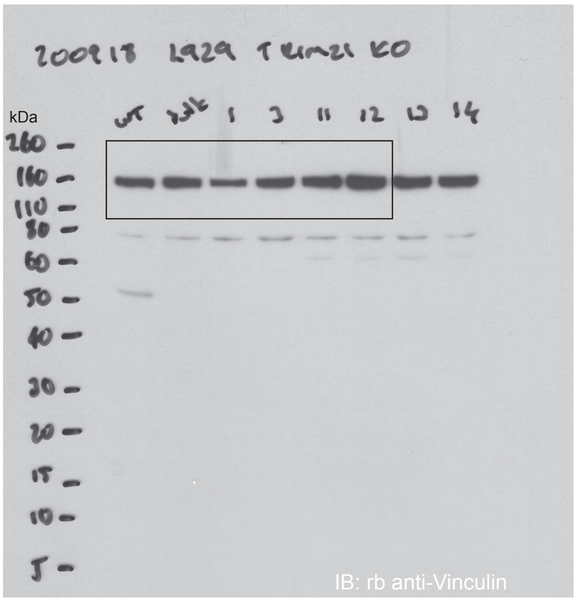

uncropped blots for panel J

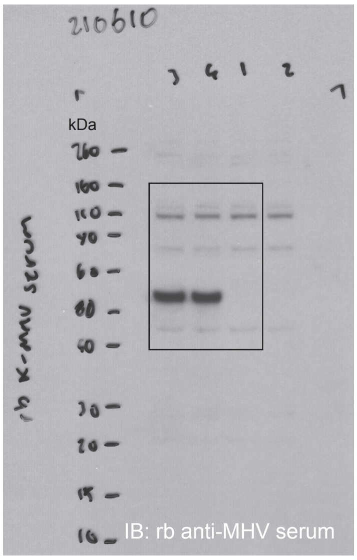

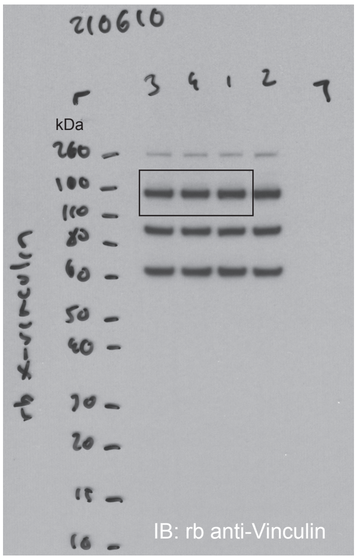

Supplement: Supplementary file 4 — Source Data for Appendix [file EMBJ-40-e108588-s003.zip › embj2021108588-sup-0005-SDataEV/embj2021108588-sup-0005-AppendixFig1.pdf]
