# Supplementary figures and images for: A functional assay for serum detection of antibodies against SARS‐CoV‐2 nucleoprotein
Source: EMBO J. 2021 Jul 29;40(17):e108588. doi: 10.15252/embj.2021108588 (PMC8408615; doi:10.15252/embj.2021108588)

Figure 1 source data

uncropped blots for panel I

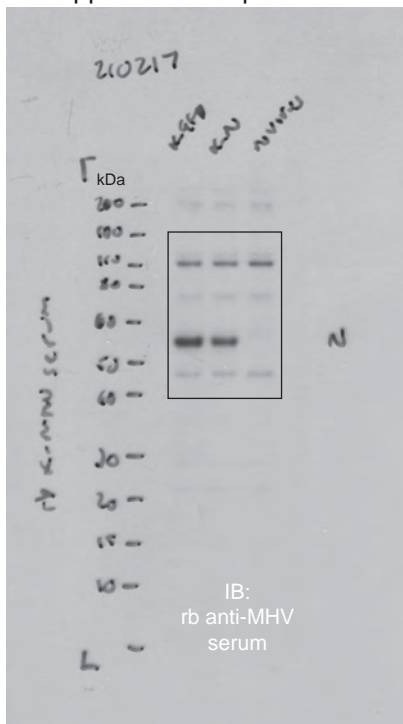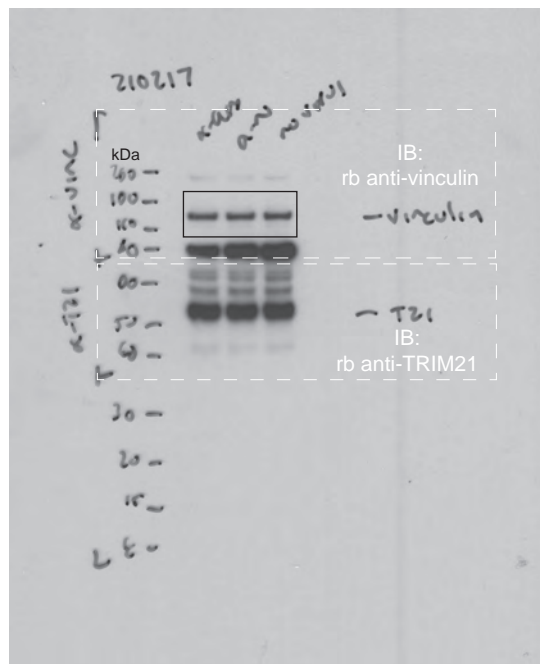

Supplement: Supplementary file 5 — Source Data for Figure 1 [file EMBJ-40-e108588-s004.pdf]

Figure 2 source data

Uncropped Li-Cor blot for 2E

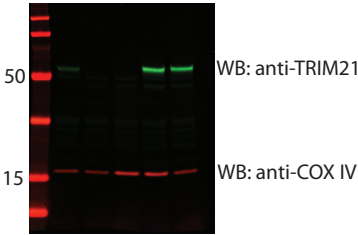

Supplement: Supplementary file 6 — Source Data for Figure 2 [file EMBJ-40-e108588-s002.pdf]
